# Retrospective Clinical Study of Advanced Pancreatic Cancer Treated With Chemotherapy and Abdominal Hyperthermia

**DOI:** 10.1200/JGO.2017.009985

**Published:** 2017-07-25

**Authors:** Yu-Fei Fan, Yuan Qin, Ding-Gang Li, David Kerr

**Affiliations:** **Yu-Fei Fan**, Beijing Yanhua Hospital; **Yuan Qin** and **Ding-Gang Li**, Beijing Haidian Hospital, Beijing, China; and **David Kerr**, University of Oxford, Oxford, United Kingdom.

## Abstract

**Purpose:**

Hyperthermia is a mechanistically plausible partner with chemotherapy, although many of the underlying molecular mechanisms of this combination treatment are not yet properly understood. Preclinical studies suggest that there is potential synergy with gemcitabine and that provides the basis for retrospective analysis of a clinical series combining these treatment modalities for patients with advanced pancreatic cancer.

**Patients and Methods:**

Twenty-nine chemotherapy-naive patients with locally advanced or metastatic pancreatic carcinoma with malignant ascites were treated with intraperitoneal cisplatin 30 mg/m^2^ and gemcitabine 800 to 1,000 mg/m^2^ intravenously on days 1, 8, and 15 every 28 days until tumor progression. Patients also received regional hyperthermia treatment (41 to 42°C) on the upper abdomen two times per week from days 1 to 21.

**Results:**

In all, 83 cycles of chemotherapy were administered and were generally well tolerated. No patients had a complete response, 13 had a partial response, seven had stable disease, and 9 had progressive disease. Mean progression-free survival and overall survival were 119 ± 61days and 195 ± 98 days, respectively.

**Conclusion:**

This study provides preliminary evidence that the treatment approach of combined systemic and intraperitoneal chemotherapy plus hyperthermia is well tolerated, is active, and has an acceptable survival profile for patients with stage IV pancreatic cancer and ascites.

## INTRODUCTION

The health burden of pancreatic cancer in China is increasing, with annual mortality rates almost equal to incidence rates, and it has been calculated that China hosts almost 20% of the world’s newly incident cases.^1^ For the subgroup of patients with advanced pancreatic cancer who present with malignant ascites, the median survival is reported to be extremely poor (63 to 81 days).^2,3^ The requirement for novel therapeutic approaches for this particularly poor prognostic group is obvious.

Hyperthermia (HT) uses high-frequency electromagnetic waves to heat tumor cells to 41° to 45°C. These increased temperatures can alter the pathophysiology of the cancer by increasing the permeability of tumor cells and facilitating cytotoxic drug diffusion, reducing and reversing multidrug resistance in tumor cells, inhibiting the repair of DNA damage, upregulating heat shock protein expression, increasing tumor antigenicity, and enhancing natural killer (NK) cell activity.^4-8^

There is some evidence to suggest that intraperitoneal (IP) administration of cytotoxic drugs generates a pharmacokinetic advantage by delivering much higher peritoneal drug concentrations compared with systemic administration. Increasing cytotoxic drug exposure within the compartment harboring the majority of the tumor burden may increase tumor response rates. This has been proven in a large, well-designed randomized trial which has demonstrated that IP administration of cisplatin confers a survival advantage for patients with ovarian cancer compared with its conventional intravenous administration.^9^

Here, we report our experience using a combination of chemotherapy (intravenous gemcitabine plus IP cisplatin) combined with regional radiofrequency thermotherapy (RFTT) as a first-line treatment for patients who presented with advanced pancreatic cancer and ascites.

## PATIENTS AND METHODS

Our results were obtained from a retrospective analysis of a protocol-driven clinical series of consecutive patients who presented to the YuanHua Hospital in Beijing, China. All patients provided written informed consent for the treatment regimen; however, because this was not a prospective clinical trial, no institutional review board approval was sought. Recruitment took place between 2007 and 2012. Patient characteristics are summarized in Table 1.

**Table 1:**
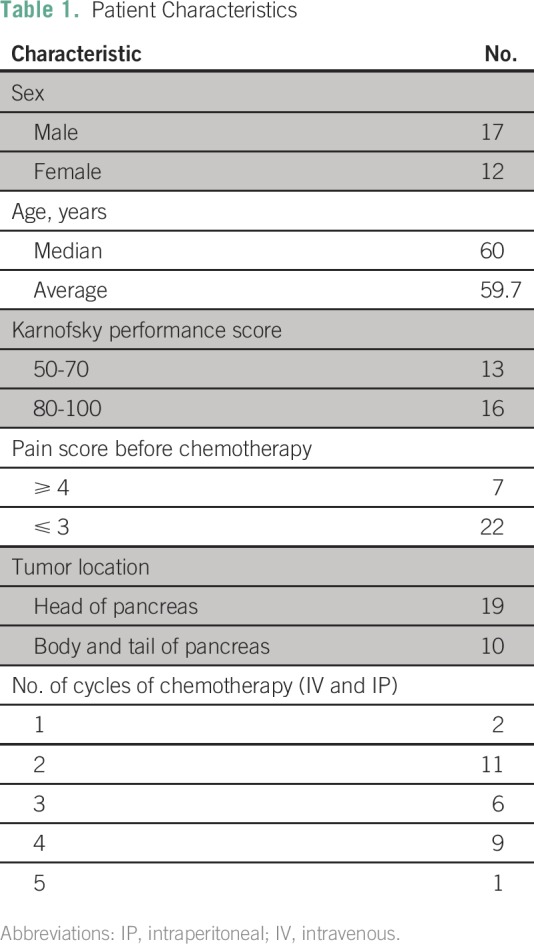
Patient Characteristics

### Treatment Procedure

After draining ascites, an indwelling peritoneal catheter was inserted under local anesthetic control. This permitted IP infusion of cisplatin 30 mg/m^2^ (in 2 to 2.5 L of normal saline over 30 minutes) on days 1, 8, and 15. Gemcitabine was administered at 800 to 1,000 mg/m^2^ intravenously by 30-minute infusion on days 1, 8, and 15 and repeated every 28 days until progression was documented. These patients also received regional hyperthermia treatment (41 to 42°C) for 1 hour on the upper abdomen twice per week for 3 weeks (days 1, 4, 8, 11, 15, and 18; Table 2).

**Table 2:**
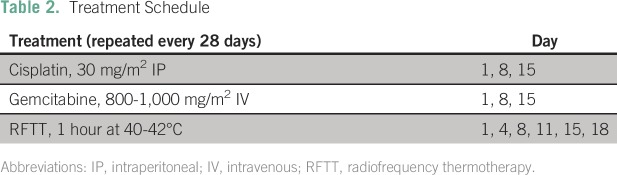
Treatment Schedule

**Fig 1:**
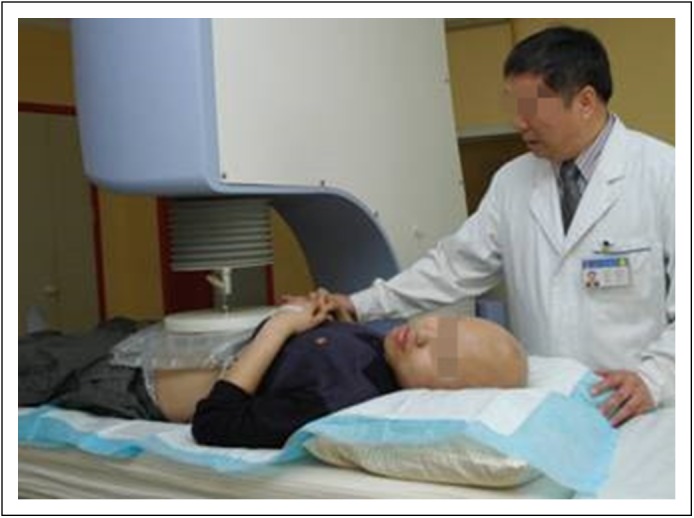
Patient being treated with abdominal radiofrequency thermotherapy.

The device used was the SR 1000 tumor hyperthermia system from Beijing Xinke Establish Science and Technology (Fig 1). The target temperature of the treated area was set at 42°C. Heat was applied through a pair of electrodes placed on opposite sides of the hepatic region. Each electrode was covered with a water pad, and a saline solution maintained at 5°C was perfused into the water pad to avoid excessive heating of skin and subcutaneous fat. Treatment time per session ranged from 40 to 60 minutes (depending on patient tolerance) at a power setting of 150 W. Blood pressure and pulse rate were monitored every 15 minutes during hyperthermia. Body temperature was measured before and after treatment.

### Main Outcome Measures

Patients were observed for evidence of response (Response Evaluation Criteria in Solid Tumors [RECIST]) by abdominal computed tomography (CT), magnetic resonance imaging (MRI), or positron emission tomography-CT (PET-CT) scans. Toxicity was recorded by using Common Terminology Criteria for Adverse Events (CTCAE v3.0), and progression-free survival (PFS) and overall survival (OS) were measured from day 1 of the first cycle of treatment. Kaplan-Meier plots were made from survival data.

## RESULTS

### Patient Characteristics

In all, 29 patients were recruited (17 male, 12 female); age ranged from 34 to 74 years, with a median age of 60 years. Primary tumors were located predominantly in the pancreatic head and neck (n = 19), with the remainder located in the pancreatic body and tail (n = 10). According to the American Joint Committee on Cancer (AJCC; 7th Edition) staging, all patients had stage IV disease. All patients were chemotherapy naive. Prechemotherapy, Karnofsky performance score (KPS) for 13 patients (44.8%) was 50 to 70, and for 16 patients (55.2%), it was 80 to 100. The 29 patients completed a total of 83 cycles of chemotherapy (range, one to five cycles; median, three cycles; Table 1)

### Efficacy Analysis

Follow-up using the most appropriate imaging technique (CT, MRI, or PET-CT) was repeated every two cycles, and all scans were reviewed by a single radiologist using RECIST criteria. There were no complete responses (CRs), 13 patients (44.8%) had partial responses (PRs), and seven patients (24.4%) had stable disease (SD). Nine patients (31.0%) had progressive disease (PD). Overall objective response rate (CR + PR) was 44.8%, and disease control rate (CR + PR+ SD) was 70.0%. Mean PFS for the whole group (n = 29) was 119 ± 61 days; the mean OS was 195 ± 98 days.

### Safety Analysis

Common toxicities included grade 2 nausea and vomiting (10.3% of patients), grade 3 or 4 thrombocytopenia (13.8%), grade 2 or 3 fatigue (13.8%), and grade 3 or 4 neutropenia (17.2%). Grade 1 or 2 fever was found in 17.2% of patients and grade 1 or 2 abdominal pain was found in 34.5% after IP cisplatin administration. Abdominal pain was improved by increasing the volume of normal saline used in IP infusion (Table 3).

**Table 3:**
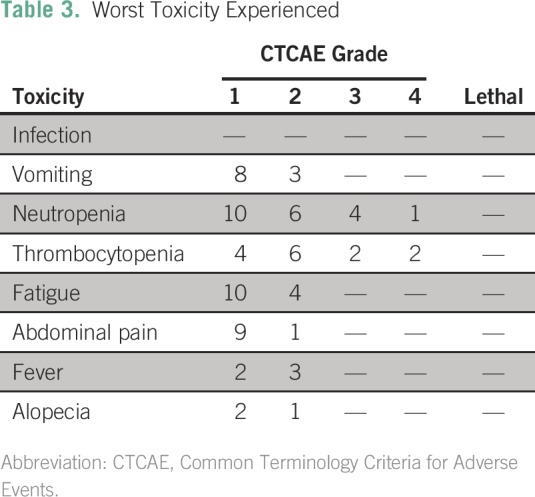
Worst Toxicity Experienced

## DISCUSSION

This small, retrospective clinical study suggests that systemic gemcitabine plus IP cisplatin in combination with RFTT was well tolerated in patients with advanced pancreatic cancer and associated ascites. This treatment approach offered a remarkable disease control rate and respectable OS in this notably refractory patient group.^2^

The standard treatment for pancreatic cancer has evolved over the last decade, moving away from single-agent gemcitabine to more complex regimens such as fluorouracil, leucovorin, irinotecan, and oxaliplatin (FOLFIRINOX). A large well-designed study has demonstrated that good-performance-score patients with advanced pancreatic cancer treated with FOLFIRINOX had improved survival (11.1 *v* 6.8 months), PFS (6.4 *v* 3.3 months), and objective response rates (31.6% *v* 9.4%) compared with single-agent gemcitabine.^10^ Only 20% of patients in this trial were found to have peritoneal tumor deposits and, generally, they had a higher performance status and perhaps better prognosis than the wider population of patients with advanced pancreatic cancer. There is increasing interest in hyperthermic IP chemotherapy (HIPEC) for treating patients with cancer who have peritoneal metastases; however, there is little clinical experience with this modality in pancreatic cancer. There are some preliminary data (n = 21) for using HIPEC in an adjuvant setting after resection of primary pancreatic cancer,^11^ suggesting that it might reduce local recurrence rates, but much larger studies are required.

The obvious limitations for our study are its size, single-center experience, lack of a control group, and the fact that data collection and analysis were retrospective rather than prospective. Nevertheless, these pilot data are sufficiently compelling to warrant further investigation, perhaps through a prospective multicenter trial that could simply aim to repeat these results or include a factorially randomized element, say with or without hyperthermia, and intravenous versus IP cisplatin administration, to better define the contribution of the individual components of this regimen.
